# Integrating shared decision-making into primary care: lessons learned from a multi-centre feasibility randomized controlled trial

**DOI:** 10.1186/s12911-021-01673-w

**Published:** 2021-11-22

**Authors:** Catherine H. Yu, Farid Medleg, Dorothy Choi, Catherine M. Spagnuolo, Lakmini Pinnaduwage, Sharon E. Straus, Paul Cantarutti, Karen Chu, Paul Frydrych, Amy Hoang-Kim, Noah Ivers, David Kaplan, Fok-Han Leung, John Maxted, Jeremy Rezmovitz, Joanna Sale, Sumeet Sodhi, Dawn Stacey, Deanna Telner

**Affiliations:** 1 Li Ka Shing Knowledge Institute of St. Michael’s Hospital, 30 Bond Street, Toronto, ON M5B 1W8 Canada; 2 Department of Medicine, University of Toronto, 190 Elizabeth Street, Toronto, ON M5G 2C4 Canada; 3 Dalla Lana School of Public Health, University of Toronto, 155 College St, Toronto, ON M5T 3M7 Canada; 4 Royal College of Surgeons in Ireland, 123 St. Stephen’s Green, Dublin, D02 YN77 Ireland; 5 School of Medicine, Queen’s University, 99 University Ave, Kingston, ON K7L 3N6 Canada; 6 Institute of Health Policy, Management and Evaluation, University of Toronto, 155 College St, Toronto, ON M5T 3M6 Canada; 7 Southlake Regional Health Centre, 596 Davis Dr, Newmarket, ON 3Y 2P9 Canada; 8 Bridgepoint Active Healthcare (Sinai Health System), 1 Bridgepoint Dr, Toronto, ON M4M 2B5 Canada; 9 Mount Dennis Weston Health Centre, Humber River Family Health Team, 2050 Weston Rd, York, ON M9N 3M4 Canada; 10 Department of Family Medicine, Women’s College Hospital, 76 Grenville St, Toronto, ON M5S 1B2 Canada; 11 University of Toronto, 1 King’s College Cir, Toronto, ON M5S 1A8 Canada; 12 North York Family Health Team, 240 Duncan Mill Rd, North York, ON M3B 3S6 Canada; 13 Markham Stouffville Hospital, 381 Church St, Markham, ON L3P 7P3 Canada; 14 Sunnybrook Health Sciences Centre, 2075 Bayview Ave, Toronto, ON M4N 3M5 Canada; 15 Musculoskeletal Health and Outcomes Research - Li Ka Shing Knowledge Institute of St. Michael’s Hospital, 209 Victoria St, Toronto, ON M5B 1T8 Canada; 16 Toronto Western Family Health Team, Toronto General Hospital Research Institute, University Health Network, 440 Bathurst St, Toronto, ON M5T 2S8 Canada; 17 School of Nursing, University of Ottawa, 451 Smyth Rd, Ottawa, ON K1H 8M5 Canada; 18 Ottawa Hospital Research Institute, 501 Smyth, Ottawa, ON K1H 8L6 Canada; 19 South East Toronto Family Health Team (Toronto East Health Network), 833 Coxwell Avenue, Toronto, ON M4C 3E8 Canada

**Keywords:** Shared decision-making, Priority setting, Patient decision aid, Interprofessional care, Diabetes mellitus, Patient education, Medical informatics, Cluster randomized controlled trial, Qualitative methods

## Abstract

**Background:**

*MyDiabetesPlan* is a web-based, interactive patient decision aid that facilitates patient-centred, diabetes-specific, goal-setting and shared decision-making (SDM) with interprofessional health care teams.

**Objective:**

Assess the feasibility of (1) conducting a cluster randomized controlled trial (RCT) and (2) integrating *MyDiabetesPlan* into interprofessional primary care clinics.

**Methods:**

We conducted a cluster RCT in 10 interprofessional primary care clinics with patients living with diabetes and at least two other comorbidities; half of the clinics were assigned to *MyDiabetesPlan* and half were assigned to usual care. To assess recruitment, retention, and resource use, we used RCT conduct logs and financial account summaries. To assess intervention fidelity, we used RCT conduct logs and website usage logs. To identify barriers and facilitators to integration of *MyDiabetesPlan* into clinical care across the IP team, we used audiotapes of clinical encounters in the intervention groups.

**Results:**

One thousand five hundred and ninety-seven potentially eligible patients were identified through searches of electronic medical records, of which 1113 patients met the eligibility criteria upon detailed chart review. A total of 425 patients were randomly selected; of these, 213 were able to participate and were allocated (intervention: n = 102; control: n = 111), for a recruitment rate of 50.1%. One hundred and fifty-one patients completed the study, for a retention rate of 70.9%. A total of 5745 personnel-hours and $6104 CAD were attributed to recruitment and retention activities. A total of 179 appointments occurred (out of 204 expected appointments—two per participant over the 12-month study period; 87.7%). Forty (36%), 25 (23%), and 32 (29%) patients completed *MyDiabetesPlan* at least twice, once, and zero times, respectively. Mean time for completion of *MyDiabetesPlan* by the clinician and the patient during initial appointments was 37 min. From the clinical encounter transcripts, we identified diverse strategies used by clinicians and patients to integrate *MyDiabetesPlan* into the appointment, characterized by rapport building and individualization. Barriers to use included clinician-related, patient-related, and technical factors.

**Conclusion:**

An interprofessional approach to SDM using a decision aid was feasible. Lower than expected numbers of diabetes-specific appointments and use of *MyDiabetesPlan* were observed. Addressing facilitators and barriers identified in this study will promote more seamless integration into clinical care.

*Trial registration* Clinicaltrials.gov Identifier: NCT02379078. Date of Registration: February 11, 2015. Protocol version: Version 1; February 26, 2015.

**Supplementary Information:**

The online version contains supplementary material available at 10.1186/s12911-021-01673-w.

## Introduction

Competing patient–physician priorities in the context of conditions with evidence-based treatment guidelines present challenges in the provision of care for complex individuals with multiple comorbidities [1]. Shared decision-making (SDM) can help prioritize treatment options and has the potential to improve patient care [2]. With SDM, patients and clinicians establish an ongoing partnership, exchange information, discuss the available options, decide which option is best, and then act on that decision [3–5]. SDM can be facilitated by the use of patient decision aids (PtDAs) [2, 3, 6] as they help frame the decision to be made. A systematic review of 105 studies found that PtDAs improved the quality of decisions and the process of decision-making, and reduced decisional conflict, but had no impact on quality of life [7]. In the trials on diabetes decision-making that were included in this review, patients who used PtDAs were more likely to change their medication. For example, Mullan et al. found that a diabetes medication choice decision aid engaged patients in their decision-making, although adherence and HbA1c did not improve [8].

Despite evidence supporting the role of SDM in complex diabetes care, many barriers make it difficult to integrate SDM and PtDAs into clinical practice. For example, patient-reported barriers include power imbalances between patients and clinicians, limited health literacy, and some patients’ denial of their condition [9]. Similarly, in our prior work, patients identified their lack of assertiveness and knowledge, limited access to their health care team, and the lack of a therapeutic relationship as barriers to SDM [1]. Clinicians struggled with decision-making when there was a disconnect between the potential goals they selected for the patient and the goals the patient set for themselves [10]. The use of an interprofessional (IP) team approach may facilitate SDM. Interprofessional care, where professionals from different disciplines collaborate to provide an integrated approach to patient care, [11] is particularly appropriate for diabetes care [12]. In diabetes care, participation by more than one profession, expanding roles, and adding new team members have been demonstrated to improve clinical outcomes [13–15] and may increase uptake of SDM [16].

Thus, we developed *MyDiabetesPlan*, a multi-component PtDA toolkit (with patient-directed and provider-directed components and point-of-care tools) that incorporates an IP approach to SDM and helps to individualize care priorities [1, 17].

Decision aids must be tested to determine their impact on patient-centered and clinical outcomes. However, there are challenges to conducting this type of evaluation, such as lack of acceptability, adherence, intervention delivery, recruitment, and retention [9]. As part of the development and evaluation of a complex intervention, the Medical Research Council recommends a feasibility and piloting phase to test procedures, estimate recruitment and retention, and determine sample size [18].

Our goals were to assess the fidelity of our intervention (i.e., if, how, and when the *MyDiabetesPlan*, toolkit was used in clinical care) as well as the feasibility of scaling our study up to a larger randomized controlled trial (RCT) to determine the efficacy and effectiveness of the toolkit to improve patient-centred and clinical outcomes.

## Methods

### Research program overview

This study is part of a multi-phased research program described elsewhere [1]. Briefly, in the first four phases we developed *MyDiabetesPlan* and conducted usability testing. The last two phases involve evaluating the effectiveness of *MyDiabetesPlan* by conducting a two-step clustered RCT and individual interviews. The primary objectives of the present study are to assess the feasibility (including cost) of conducting a larger clustered RCT (Objective 1) and to evaluate the fidelity of the intervention (Objective 2). In this manuscript we report these results according to the CONSORT statements [19] (Additional file 1: Figure S1 Appendix 1 CONSORT checklist).

### Study design

The RCT feasibility study protocol is reported elsewhere [20]. Briefly, we conducted a two-step parallel clustered RCT with a one-to-one allocation ratio. The first step was provider-directed (*MyDiabetesPlan* was delivered to physicians, nurses, dietitians, or pharmacists); the second step (six months later) was provider- and patient-directed (*MyDiabetesPlan* was also delivered to the patient). In prior usability and feasibility testing, we found that patients required assistance completing components of *MyDiabetesPlan* the first time [21]. We selected an IP approach given its effectiveness in diabetes care [15] and to facilitate uptake of SDM [1]. Patients and health care providers who participated in the study were asked to complete three questionnaires (baseline, interim [at six months], and final).

### Setting, participants, and recruitment

Family health teams were recruited from across Southern Ontario. In Ontario, health care services are publicly funded benefits with no co-payment; medications are not publicly funded benefits except for those over the age of 65 or on social assistance. Several models of primary care delivery exist, with approximately 25% of services delivered via family health teams [22]. In these groups, physician payment is primarily via capitation, while non-physicians are salaried. Groups without IP staff (nurse, dietitian, or pharmacist) or electronic medical records (EMR) were excluded. All physicians from each group were invited to participate. Once participation of the group and individual physicians in the group was confirmed, the research team worked with each of the sites to develop site-specific protocols to identify, screen, and contact patient participants, provide site-specific electronic medical record (EMR) training and access, provide privacy and research ethics training, and obtain research ethics approval.

Patients living with diabetes and at least two other comorbidities (heart disease, stroke, hypertension, cancer, chronic lung disease, arthritis, inflammatory bowel disorders, and urinary incontinence) were identified from each consenting physician’s practice by the research team or the site’s data manager using the EMR; this constituted the *pool of potentially eligible patients*. We selected patients with multiple comorbidities because this population, with competing health priorities, may derive particular benefit from a SDM and goal-setting tool. Patients were excluded if they did not speak English, had documented cognitive deficits, were unable to provide informed consent, had limited life expectancy (less than one year), or were not available for follow-up; those remaining constituted the *pool of eligible patients*. Evaluation of the exclusion criteria was done by chart review and review by the patient’s physician. From this pool, patient participants were randomly selected.

### Randomization

Family health teams were simultaneously randomized and allocated by a biostatistician to either intervention or control clusters using computer-generated randomization in a one-to-one ratio. We created a random computer-generated list of all eligible patients from each cluster. The first 40 patients from this list were invited to participate; additional invitations to the subsequent 25 participants were sent if we did not achieve participation of 25 patients from the initial group.

### Intervention

*MyDiabetesPlan* is an interactive online PtDA written at a Grade 8 English literacy level [23], based on the IP-SDM framework [24], which follows the International Patient Decision Aids Standards criteria [23]. *MyDiabetesPlan* development has been described previously [1, 23] (Additional file 2: Table S1 TIDieR Checklist). *MyDiabetesPlan* obtains the patient’s cardiometabolic and psychosocial profile, determines the patient’s general priorities of care, elicits their goals and outcomes for diabetes, outlines potential evidence-based treatment strategies as well as their risks and benefits, and synthesizes these and the patient’s values and selected strategies into an action plan. *MyDiabetesPlan* was contextualized to each site, taking into account the roles, responsibilities, and processes of care of the health care team.

*MyDiabetesPlan* and its accompanying implementation toolkit consisted of the online decision aid (available at mydiabetesplan.ca), a one-page provider enabler (a laminated sheet summarizing the purpose and flow of the decision aid, with instructions and accompanying screenshots), a similar one-page patient enabler, and brief training videos for both providers and patients. In Step One (provider-directed intervention phase), a research team member conducted a 30-min individual training session with each of the health care providers (HCPs) in the practices randomized to the intervention group. In Step Two (provider- and patient-directed phase), a link to *MyDiabetesPlan* was emailed to eligible patients. Intervention sites also participated in a group didactic session regarding SDM and one-on-one orientation sessions with email and telephone follow-up at study onset, followed by quarterly debriefing sessions, in both individual and group formats.

### Control

A paper copy of the executive summary of the Diabetes Canada (DC) clinical practice guidelines and a postcard outlining online clinical information resources were distributed to each of the HCPs in practices randomized to the control group. After six months, copies of a DC patient education pamphlet regarding diabetes self-management and a postcard outlining additional online patient resources were mailed to eligible patients. These provider- and patient-directed guideline dissemination tools (not incorporating SDM) were also publicly accessible from the DC guidelines website (guidelines.diabetes.ca).

### Objective 1 (feasibility of conducting a trial)

#### Outcome measures

We assessed the following predetermined outcomes [19]: recruitment period length, recruitment response rate, participant retention rate, questionnaire completion rate (interim and final), and resource use (personnel and funds; divided into resources required for delivery of the intervention and resources required for evaluation). The estimated sample size was 112 individuals for the primary outcome of decisional conflict [10]. We deemed the study to be feasible if we were able to complete patient recruitment within a 12-month period and attained a questionnaire response rate of 75% or higher. We defined recruitment period length as the time between when we invited the first family health team and when we recruited the final patient participant. Recruitment response rate was the total number of patients who agreed to participate in the study (signed consent and completed baseline questionnaire), divided by the total number of patients invited by study personnel to participate. Participant retention rate was the number of patients who completed the study (underwent intervention or control, or completed a follow-up questionnaire), divided by the total number of patients who agreed to participate in the study. Interim questionnaire completion rate was the number of patients who completed the second questionnaire, divided by the total number of participating patients. Final questionnaire completion rate was defined as the number of patients who completed the final questionnaire, divided by the total number of participating patients. We collated the personnel time and funds required to identify and recruit eligible patient participants; remind them about appointments, intervention use, or questionnaire completion, by telephone, mail, or in person; and manage the study data. We did not include the time or resources required for *MyDiabetesPlan* development, activities outside the trial (e.g., post-trial interviews), or analysis (e.g., statistician time).

#### Data sources and collection

Data sources and the method of collection for each outcome measure are indicated in Table 1.

**Table 1:** Method and sources of data collection

Outcome	Outcome measure	Data source	Method and timing of data collection
*Objective 1*			
Feasibility of trial conduct	Recruitment period length	Trial conduct logs [10]	Maintained prospectively by study personnel
	Recruitment response rate		
	Participant retention rate		
	Questionnaire completion rate		
	Resource use (personnel and funds)	Financial account summaries	Produced at study completion by the financial analyst affiliated with the principal investigator’s institutional research office Corroborated by expense and personnel logs maintained prospectively by study personnel
*Objective 2*			
Intervention fidelity	Total and mean number of appointments over study period	Trial conduct logs	Maintained prospectively by study personnel
	Number of times *MyDiabetesPlan* was used	Website usage logs	
	Number of plans completed		
	Time needed to complete *MyDiabetesPlan*	Audiotapes of clinical encounters	Consent obtained from participants at time of clinical encounter during study period Entire clinical encounter audiotaped then transcribed verbatim
	Time needed to complete each section		
	Integration of *MyDiabetesPlan* into clinical encounter		
*Additional patient-centred and HCP-centred outcomes*			
Socio-demographic information	Providers: gender, duration in practice, practice load, academic/community	Online or mailed survey	At study start
	Patients: age, gender, ethnicity, age at diagnosis, comorbidities, smoking status, educational attainment, annual income		
Patient-centred outcomes	Decisional conflict [25]	Online or mailed survey	Completed by patient at baseline, at six months, and at study completion for a total of three data points over a 12-month period Reminded twice by email or telephone at two-week intervals to complete the questionnaires
	Diabetes distress [26]		
	Health-related quality of life [27]		
	Chronic illness care [28]		
HCP-centred outcomes	Intention to engage in IP-SDM [29]	Online or mailed survey	Completed by HCP at baseline, at six months, and at study completion for a total of 3 data points over a 12-month period Reminded twice by email or telephone at two-week intervals to complete the questionnaires

#### Analysis

We calculated basic frequencies and conducted descriptive statistical analyses of the above metrics for all sites combined as well as for each individual site.

### Objective 2 (intervention fidelity)

#### Outcome measures

We recorded the total and mean number of appointments over the study period as well as *MyDiabetesPlan* usage as measures of intervention fidelity. Specifically, we assessed the number of times *MyDiabetesPlan* was used over the study period (defined as number of logins), the number of plans completed (defined as the number where an action plan was present), the time needed to complete *MyDiabetesPlan* (defined as the duration of time from login to plan completion), and the time needed to complete each section (part one (Where I am now), part two (Where I want to be), part three (How I will get there), part four (What I’m going to do to get there)).We assessed how *MyDiabetesPlan* was integrated into the clinical encounter by analyzing audiotapes of clinical encounters.

#### Data sources and collection

Data sources and the method of collection for each outcome measure are indicated in Table 1.

#### Analysis

We calculated basic frequencies and conducted descriptive statistical analyses of the above metrics for all sites combined as well as for each individual site. Transcripts of clinical encounters were analyzed inductively by at least two coders [30, 31] with the lens of understanding how *MyDiabetesPlan* was integrated into the clinical encounter [32, 33]; our goal was to better understand how clinicians and patients integrated this web-based decision aid into clinical care, as well as barriers and facilitators to its integration. A coding framework was developed iteratively and validated with research team members, including patient knowledge users [34]. NVivo 8 (QSR version 9.2.81.0) was used to facilitate data analysis.

## Results

### Objective 1: feasibility of conducting a trial

#### Recruitment period length, recruitment response rate

**Fig. 1:**
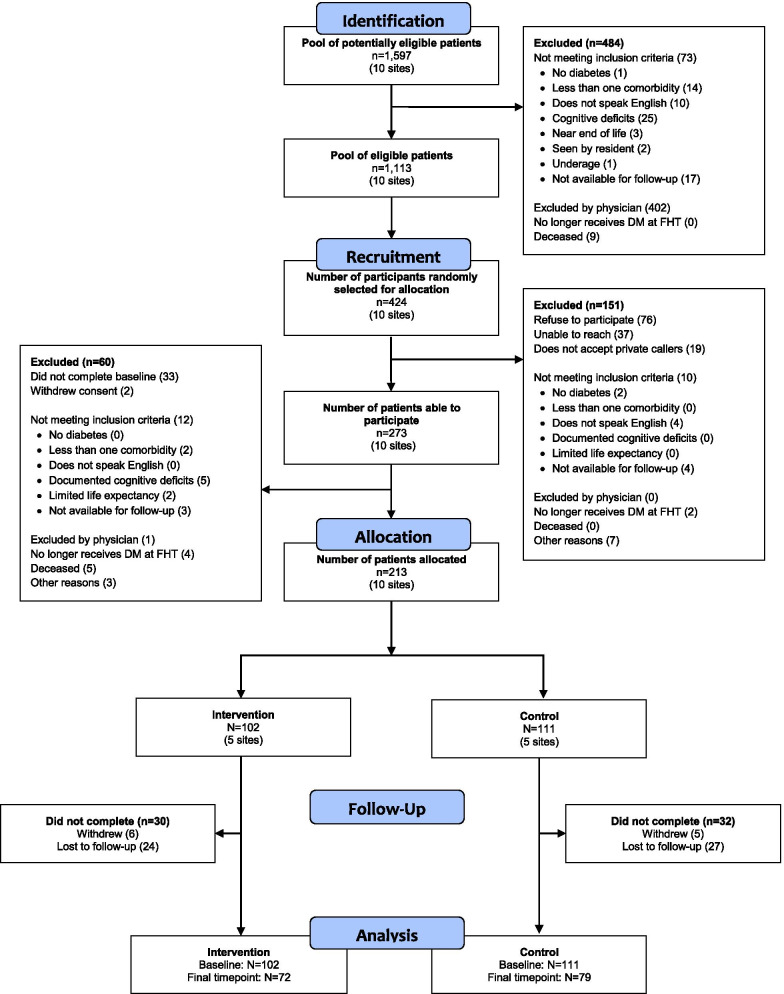
CONSORT flow diagram. *Abbreviations*: DM, diabetes mellitus; FHT, family health team

Recruitment metrics are summarized in Fig. 1. We recruited 10 family health teams over the first 12 months (December 2014–November 2015), then we recruited patients over the next 10 months (December 2015–September 2016), for a total recruitment period of 22 months. We identified a total of 1597 potentially eligible patients through searches of the EMRs of the 10 family health teams. From this pool, we identified through chart review 1113 patients who met the eligibility criteria. We then randomly selected and contacted 424 patients. Of these, 274 were able to participate; 61 patients were excluded by further screening, leaving 213 patients, for a recruitment response rate of 50.1%.

#### Participant retention rate and questionnaire completion rate

Of these 213 patient participants, 151 completed the study, for a participant retention rate of 70.9%. A total of 130 and 151 patient participants completed the interim and final questionnaires, respectively, for questionnaire response rates of 61.0% and 70.9%, respectively.

#### Resource use (personnel and funds)

A total of 6217 hours of personnel time was required to implement the study protocol. Not including funds required for personnel, $6104 CAD was used for study-related materials. Tables 2 and 3 outline the breakdown of resource use. The cost per participant retained was 41 hours and $40.42 CAD over the study period.

**Table 2:** Personnel costs: breakdown of hours used to implement study protocol

Personnel costs	Hours used
*Participant contact*	*5383*
Phone calls	5376
Mailing	5
Emailing	2
Facilitating delivery of intervention	7
Clinician training	18
Data management	73
*Intervention evaluation*	*736*
Conducting usability sessions	69
Transcription	219
Usability analysis	329
Post-RCT analysis	119
Total hours used	6217

**Table 3:** Materials, services, and supplies costs: breakdown of funds used for study-related materials

Materials, services and supplies costs	Funds used (CAD)
*Study-related materials*	
Mailing	$4494
Teleconference meetings	$646
Data and information technology platforms	$244
Study honouraria	$720
Total funds used	$6104

### Objective 2: Intervention fidelity

#### MyDiabetesPlan use

In total, 179 appointments were conducted with the 102 patient participants in the intervention group over the study period, resulting in a mean of 1.8 appointments per participant. Fourteen, 42, and 27 participants attended one, two, and three appointments, respectively. *MyDiabetesPlan* was used 170 times over the study period, or 1.7 times per participant. There were a total of 140 action plans completed, or 1.30 plans per participant. Twenty-five, 35, and 15 participants completed the plan once, twice, and more than two times, respectively; 32 participants did not complete the plan (16 of these participants started but did not complete the plan).

Fifty-six clinical encounters using *MyDiabetesPlan* were audiotaped; the distributions of these encounters among sites and by appointment number are indicated in Additional file 2: Table S2. Completion metrics are indicated in Table 4.

**Table 4:** Time required to complete *MyDiabetesPlan* and its components during initial and follow-up visits

Time needed to complete (minutes: seconds)	Mean (SD)	Range	
		Minimum	Maximum
*Initial visit*			
Entire action plan	36:36 (14:02)	17:02	1:06:04
Part 1	22:07 (9:22)	4:26	33:32
Part 2	02:07 (1:18)	0:08	5:15
Part 3	06:33 (04:30)	1:06	16:18
Part 4	05:50 (04:22)	0:19	11:36
*Follow-up visit*			
Entire action plan (review)	16:29 (11:46)	1:36	54:52

#### Integration of MyDiabetesPlan into the clinical encounter

We analyzed 58 audiotaped clinical encounters during which clinicians used *MyDiabetesPlan*. We organized the data into four categories; representative quotes are listed in Additional file 2: Table S3.

#### Clinicians’ approach to *MyDiabetesPlan*

Clinicians commonly viewed the decision aid as a medium to exchange information and engage patients in diabetes self-management. Its questions gave clinicians the opportunity to learn about their patients and gather history. Such information provided the basis for effective discussions beginning with an overview of diabetes self-management, followed by patient-specific dialogue regarding health status, laboratory results, and current medications.

We identified two key strategies that clinicians used that facilitated use and completion of *MyDiabetesPlan* during the appointment: engaging the patient in their care and providing information. For example, one clinician discussed their own struggles with routine physical activity to develop a rapport with the patient and engaged the patient in their personal physical activity goals, demonstrating a commitment to lead by example. Other clinicians provided an explanation and rationale to help the patient understand their test results and gain a better picture of their blood sugar control. Some providers used humour to engage patients such as teasing about a “magic pill” or discussing the disappointments of a sports team. Addressing barriers to adherence specific to each patient, individualizing *MyDiabetesPlan* on the basis of patient circumstances, and providing encouragement and positive feedback were other strategies used to engage the patient.

Provision of information to the patient was an important precursor and component of *MyDiabetesPlan* use. Clinicians provided an overview of general diabetes management to contextualize *MyDiabetesPlan*, then moved on to provide specific details regarding that patient’s own health status, and then contrasted the two to highlight potential areas to work on. Finally, they provided additional informational resources (such as handouts) to reinforce and finalize the plan.

The degree of *MyDiabetesPlan* integration was variable and dependent on clinician engagement. Some clinicians appeared more eager than others to use *MyDiabetesPlan* during appointments and started by introducing *MyDiabetesPlan* in a positive light. Some clinicians demonstrated enthusiasm about its use by summarizing and printing the patient’s action plan at the end of the visit and seeking the patient’s perspective on the effectiveness of *MyDiabetesPlan*. Others were less enthusiastic, starting with their own diabetes-specific agendas, returning to the patient’s concerns once this discussion was complete, and indicating that the clinician themselves had to leave but would “try and get through this (completion of *MyDiabetesPlan*).”

#### Patients’ response to *MyDiabetesPlan*

Based on transcripts of the taped clinical encounters, patients’ experience with *MyDiabetesPlan* was generally positive, with most engaged with its use. Its use allowed them to express their beliefs and experiences and direct their care. For example, when asked about how important *MyDiabetesPlan* was to achieving their goal, one patient said to the clinician that she was motivated by her fear of “going on insulin” and her belief that starting insulin would liberalize her dietary choices and result in her eating more cake. Understanding this patient’s beliefs and perspectives allowed the clinician to tailor education and management plans to the patient. *MyDiabetesPlan* offered patients the opportunity to direct goal setting; some patients completed *MyDiabetesPlan* independently, enabling them to take control of their own care.

#### Challenges with integrating *MyDiabetesPlan* into clinical care

Though *MyDiabetesPlan* was successfully integrated into the clinical appointment in most cases, we identified barriers to optimal integration. We categorized these into clinician-related, patient-related, and other (technical) factors.

Clinician-related factors included reluctance to use a separate online tool and change their usual approach to conducting a diabetes appointment (such as format and organization). Some clinicians felt uncomfortable about asking certain questions in *MyDiabetesPlan*, such as asking for the name of a potential partner who provides social support. Some clinicians felt limited by a perceived inflexibility of *MyDiabetesPlan* as they wanted to select several diabetes management goals and/or strategies for their patients; *MyDiabetesPlan* limited them to selecting one goal and strategy. This did not fit with how clinicians normally would approach and discuss goal setting with their diabetic patients. Finally, clinicians thought that some of the questions were redundant, such as being asked about cholesterol control on the opening screen to assess whether *MyDiabetesPlan* use is relevant to the patient and again later in the comorbidity section.

Patient-related factors included patient complexity, language and cultural barriers, and difficulty with interpreting, and thus answering, *MyDiabetesPlan* prompts. For instance, some patients considered walking to be moderate-intensity physical activity, whereas *MyDiabetesPlan* classifies walking as mild-intensity exercise. Other patients had difficulty with navigating *MyDiabetesPlan* independently as it was not something they were used to doing and/or they were not comfortable using online programs. Similar to clinicians, patients also felt that there was a lack of flexibility in *MyDiabetesPlan.* For example, some felt that the strategies suggested were limited and did not match what they would have wanted to select, such as reducing meal portion sizes rather than choosing certain types of foods as suggested by *MyDiabetesPlan*.

Technical barriers external to *MyDiabetesPlan* included browser-specific errors and the requirement to log into *MyDiabetesPlan* (for example, the clinician accidentally using a different patient’s *MyDiabetesPlan* account).

#### Facilitators to integrating MyDiabetesPlan into clinical care

Engaging the patient in their care via effective communication skills was a common strategy that enabled clinicians to complete *MyDiabetesPlan*. Some clinicians were more flexible with how they used *MyDiabetesPlan* and instead of reading it verbatim would use open-ended questions, such as asking about a patient’s typical meal rather than the number of grain servings per day, and extrapolate answers from patient responses. Similarly, they would use earlier questions from *MyDiabetesPlan* to facilitate discussion, thus streamlining the appointment. Some clinicians combined *MyDiabetesPlan* use with standard of care to address patient concerns, by using prompts from *MyDiabetesPlan* as opportunities to provide patient education. For instance, when answering the *MyDiabetesPlan* question about blood sugar control, a clinician addressed the patient’s concerns about higher morning blood sugars. Clinicians also helped patients interpret results from *MyDiabetesPlan* on the basis of information the patient had previously entered and taught the patient how to use *MyDiabetesPlan* independently for the next visit.

It was evident that MyDiabetesPlan became easier to use during subsequent visits. Many clinicians used the patient’s previously completed action plan as a prompt to discuss goal-setting and/or goal achievement, providing patients the opportunity to modify their goal and/or strategies on the basis of progress and/or barriers. Revisiting the plan was an important opportunity to update current medications and review the health beliefs of the patient and the barriers they were experiencing. Increased familiarity with *MyDiabetesPlan* questions enabled clinicians to identify and correct misinformation. Prior completion of the plan by the patient had a similar effect, allowing *MyDiabetesPlan* to be more easily integrated into the usual diabetes appointment.

## Discussion

We found that conducting an RCT of *MyDiabetesPlan* was feasible: we completed patient recruitment within 12 months and achieved a questionnaire completion rate of 71% (although this is less than our a priori goal of > 75%, we were still able to achieve our target sample size). This was at a cost of 41 personnel hours and $40 CAD per participant completing the study. With respect to the feasibility of integrating *MyDiabetesPlan* into clinical care, there was a lower-than-anticipated number of diabetes-specific appointments, and only 50.1% of patients completed *MyDiabetesPlan* twice as planned. Health care providers and patients used different approaches to integrating *MyDiabetesPlan* into the appointment, characterized by rapport building and individualization. Barriers to use included clinician-related factors (such as reluctance to change their approach to diabetes appointments and discomfort with asking certain questions), patient-related factors (such as computer literacy), and technical factors. Facilitators of use included a therapeutic rapport, clinician flexibility in reframing questions and responses, patient engagement, and a pre-completed *MyDiabetesPlan* (either by the patient before the appointment, or in prior appointments).

The United Kingdom National Health Service conducted a cost analysis of projects funded by the Health Technology Assessment Programme. In 2003, the mean annual research cost per patient ranged from £24 to £4476, with a mean of £3228 [35]. Even accounting for currency exchange and inflation with time, our estimates are modest. Moving forward, if we consider scaling up this study to detect differences in clinical outcomes, a sample size of 393 would be required to detect a difference in HbA1c of 0.20% (assuming a standard deviation of 0.98 [36], two-tailed α = 0.05, β = 0.20) [37]. Thus, a large-scale RCT powered for clinical outcomes would require 15,320 personnel hours and $14,554 CAD over the study duration. Given that the majority of expenses incurred were due to recruitment costs, more efficient methods of recruitment as well as novel trial designs could be adopted to increase the feasibility of similar studies. For example, leveraging technology-enabled recruitment strategies such as social media (Facebook, Instagram, Craigslist) and research registries may be more efficient and cost-effective than using traditional recruitment strategies such as print and clinic-based strategies, marketing firms and media [38]. Use of Bayesian and adaptive trial designs [39, 40] could be another strategy by which trials could be conducted more efficiently (both operationally and analytically) while enhancing generalizability. For example, given the potential for differential efficacy of IP-SDM depending on contextual factors (patient, HCP, clinical context), a population-enrichment design, wherein a priori selected non-performing subgroups are eliminated at interim analysis, would help verify prospectively which contextual factors predict intervention effectiveness; subsequent enrolment can focus on the other subgroups [39].

We observed less-than-anticipated attendance at primary care appointments, and consequently, *MyDiabetesPlan* use “as planned” (minimum of two times to ensure longitudinal use and follow-up) was only 50%. This happened despite our pre-study consultation with HCP and patient knowledge users about the feasibility of quarterly appointments [1]. It highlights not only the importance of a pilot phase to assess “real-world” feasibility [18] but also the dependence—and interaction—of our intervention on “usual care” and the implications of this relationship for future integration of *MyDiabetesPlan* into clinical care. Literature suggests that only 27.6% to 34.4% of physicians conduct two or more comprehensive diabetes visits per patient per year, corroborating findings from our trial [41]. Barriers to primary care attendance included medical factors (such as feeling unwell, hospital admission, or resolution of symptoms), appointment system factors (such as forgetfulness or confusion about appointment time), and other logistical factors (such as traffic or oversleeping) [42]. Addressing these factors, which overlap with those affecting chronic disease management, may be a necessary co-intervention to optimize integration of *MyDiabetesPlan* into care. Alternative models of integration to circumvent dependence on usual clinical care include preconsultation aids, peer coaches [43], and virtual consultation [44], though these methods are not without their limitations.

Finally, our findings regarding integration of *MyDiabetesPlan* into clinical care highlight the importance of therapeutic rapport, effective communication skills, and clinician flexibility in overcoming barriers to integration, such as the perceived prescriptive nature of *MyDiabetesPlan*, the duration of time to complete *MyDiabetesPlan,* and patient computer literacy. This supports previous reports regarding the importance of HCP training in SDM [45] and communication skills [46] in improving uptake of SDM into practice. In our study, we conducted a small group didactic session regarding SDM, one-on-one orientation sessions with email and telephone follow-up with each clinician at study onset, followed by quarterly debriefing sessions, in both individual and group formats. However, we did not implement formal communication skills training as part of our intervention. Barriers associated with the time required to complete *MyDiabetesPlan* initially may be overcome by optimizing provider engagement (for example, implementing training and appropriate framing of time requirements to address expectations [47]) and refining *MyDiabetesPlan* to enable more clinician autonomy and patient engagement (for example, pre-completion of certain components on tablets in the waiting room [48], use of health coaches [49], and additional usability testing to ensure compatibility with patient computer literacy [50]).

Our study limitations include a low recruitment rate, lower than anticipated use of *MyDiabetesPlan*, usability issues associated with *MyDiabetesPlan* use, lack of generalizability to primary care settings with no interprofessional teams, and the timing of the study conduct. We hypothesize that our low recruitment rate was secondary to the vulnerable nature of our study population: patients with diabetes and two other comorbidities may find it challenging to participate in research studies [51], and this was reflected in our reasons for exclusion (Fig. 1. CONSORT flow diagram). While our recruitment rate was 50.1%, this is comparable to other studies [52] and because of our recruitment protocol we were still able to attain our target sample size. Despite the fact that we had lower than anticipated use and issues with usability, the study gave us important insights regarding better integration into clinical care. Although these findings will not be generalizable to solo practitioners, we selected an interprofessional team approach for our intervention given its demonstrated effectiveness in diabetes care [15]. Although the study was conducted five years ago, the findings are still relevant given the increasing use of eHealth technologies for diabetes care [53], and particularly given the increasing virtualization of primary diabetes care that occurred with the COVID pandemic [54].

Our study strengths include its high retention rate, comprehensive data collection, use of mixed methods, dual coding of clinical encounter transcripts and triangulation of qualitative with quantitative results [30, 55, 56], and research team composition (knowledge users and experts in shared decision-making, knowledge translation, information technology, primary care diabetes, and qualitative and quantitative research methods).

## Conclusions

IP-SDM can help to focus the care of patients with multiple comorbidities but has not been widely adopted into clinical practice. By assessing intervention use and fidelity, including barriers and facilitators to implementation, this study provides useful insights that can be leveraged to promote more complete and seamless integration of IP-SDM into clinical care, such as the importance of therapeutic rapport and communications training, and the potential role of adjunctive patient supports.

## Supplementary Information

**Additional file 1:** The CONSORT Checklist that outlines to report information included in a randomised trial.

**Additional file 2:** Compilation of tables that describe: MyDiabetesPlan's intervention components via TIDieR Checklist (S1 table); the division of audiotaped clinical encounters by sites and type (S2 table); and representative quotes from clinical encounters (S3 table).

## Data Availability

All quantitative data generated or analysed during this study are included in this published article [and its supplementary information files]. The qualitative datasets used and/or analysed for the second component of objective two "Integration of *MyDiabetesPlan* into the clinical encounter" are available from the corresponding author on reasonable request.

## Abbreviations

DC: Diabetes Canada
HCP: Health care provider
IP: Interprofessional
PtDAs: Patient decision aids
SDM: Shared decision-making
