# Supplementary material for: Integrating shared decision-making into primary care: lessons learned from a multi-centre feasibility randomized controlled trial
Source: BMC Med Inform Decis Mak. 2021 Nov 22;21:323. doi: 10.1186/s12911-021-01673-w (PMC8609876; doi:10.1186/s12911-021-01673-w)
Supplement: Supplementary file 2 — Additional file 2: Compilation of tables that describe: MyDiabetesPlan's intervention components via TIDieR Checklist (S1 table); the division of audiotaped clinical encounters by sites and type (S2 table); and representative quotes from clinical encounters (S3 table). [file 12911_2021_1673_MOESM2_ESM.docx]

Integrating shared decision making into primary care: Lessons learned from a multi-centre feasibility randomized controlled trial: Additional Files

**Table of Contents**

- [Additional File 2: Table S1. MyDiabetesPlan– Detailed description of intervention components](#S2) (TIDieR Checklist)
- [Additional File 3: Table S2. Audiotaped clinical encounters by site and type.](#S3)
- [Additional File 4: Table S3. Representative quotes from clinical encounters characterizing the use of *MyDiabetesPlan* in clinical care](#S4)

**Additional File 2: Table S1.** MyDiabetesPlan– Detailed description of intervention components (TIDieR Checklist)

| **Intervention component** | **Description** | **Materials/Procedures** | **Provider** | **Mode of Delivery/Location** | **When/How much** | **Tailoring, modification, fidelity** |
| --- | --- | --- | --- | --- | --- | --- |
| Small group sessions | Consisted of:   - Initial 60-minute interactive session regarding shared decision-making, - Quarterly 60 minutes debriefing sessions | Powerpoint presentation | Facilitated by a member of the research team with expertise in shared decision-making professional development | Face-to-face, at clinical site | At study start and quarterly thereafter, scheduling permitting for a total of 2-4 sessions per site | Tailored to site-specific diabetes care delivery protocols and standards, as well as site progress with respect to MyDiabetesPlan use |
| Individual training session | Consisted of:  30 minute training session between a research coordinator and provider.  One-on-one orientation sessions with email and telephone follow-up with each clinician at study onset, with debriefing sessions | Clinic computer, training script, | Facilitated by a member of the research team | Face-to-face at in HCPs clinic room, email, telephone | At study start | Tailored to site-specific clinic flow, electronic medical record and browser availability |
| Training Video | Consisted of:  A demonstration of how to use MyDiabetesPlan.  2 versions: one for providers and one for patients | Developed, reviewed, and narrated by an endocrinologist and research team | n/a | Mail, email (via URL link) | Video was tailored to guide the provider on use of the decision aid. | Standard across all intervention sites |
| How to card | Consisted of:  A summary guide describing the function of the decision aid and practical instructions on how-to-use.  2 versions: one for providers and one for patients | Developed, reviewed, and written by an endocrinologist and research team | n/a | Mail, email (laminated hard copy and/or PDF) | Instructions and images were tailored to guide the patient on use of the decision aid. | Standard across all intervention sites |
| MyDiabetesPlan | Online decision aid designed to facilitate goal setting and SDM, consisting of 4 parts:  **Part 1 - Where I am now**  My diabetes ‘vital signs’, diabetes history, current lifestyle (physical activity, eating habits) and medications, and personal supports and challenges  **Part 2 – Where I want to be**  Goal selection and motivation of why you chose this particular goal.  **Part 3 – How am I going to get there**  Strategies to promote the achievement of selected goal in based on individual descriptive statistics  **Part 4 – What I am going to do to get there** Summary of Action Plan, additional resources, and follow-up appointment | IPSDM toolkit contextualized to each site, and integrated based on HCP team roles, responsibilities, and processes in usual care. | Patients and HCPs part of their usual diabetes care team(RN, RD, pharmacist, MD) | 1. By participant themselves, then reviewed with HCP during appointment, 1-on-1 2. By participant with family members, then reviewed with HCP during appointment, 1-on-1 AND/OR with more than 1 HCP together 3. Together with HCP 1-on-1 AND/OR with more than 1 HCP together   Location: At regular diabetes clinical appointment (primary care site). Some participants have the option to complete Part 1 of the intervention at home prior their regular appointment. Computer with IE7+, Firefox or Chrome is needed  Action Plan can be saved as PDF and printed as a summary sheet | Intervention was delivered across a period between 6-12 months, with minimum 2 sessions (one initial visit, one subsequent visit) | Tailoring: Intervention was planned to be adapted to clinical flow and patient use.  Modifications: A goal tracking achievement marker was included during the course of the study. Prior visiting the MDP in a subsequent visit, users were prompted with the achievement tracker that reminds them of their goals and strategies from their previous appointment. This marker helps serve as a guide and motivator, and acts as a stepping stone to revisit the MDP in each subsequent visit.  Fidelity:  Trial conduct logs and website usage logs in order to assess intervention fidelity.  # of times MDP was: logged in, used with plan created, time needed to complete MDP, and clinical encounter integration. |

**Additional File 3: Table S2.** Audiotaped clinical encounters by site and type.

| **Site** | **# of Appointments Total** | **Initial Visits** | **Subsequent Visits** |
| --- | --- | --- | --- |
| **Intervention** | |  |  |
| 1 | 16 | 4 | 12 |
| 2 | 13 | 1 | 11 |
| 3 | 6 | 1 | 5 |
| 4 | 8 | 3 | 5 |
| 5 | 7 | 3 | 4 |
| **Control** | |  |  |
| 6 | 7 |  |  |
| 7 | 1 |  |  |

**Additional File 4**: **Table S3.** Representative quotes from clinical encounters characterizing the use of *MyDiabetesPlan* in clinical care

| Theme | Subtheme |  | Quote |
| --- | --- | --- | --- |
| 1) Clinician’s approach to *MyDiabetesPlan* | a) Engaging the patient in their care | By sharing own experiences | Pt: Yeah, yeah no I have to do that and make sure I get outside at lunch time  HCP: Even if it’s a cold day, just go on out, take a breath, and I’m gonna be honest with you [Name of Pt] I can sit in this chair and counsel with you, I just had lunch in my office. […] I know exactly where you’re coming from, it’s a tough thing to do but I think there comes a point that we have [emphasis] to [sigh] support each other. […] So I can relate to that. *[HCP32 (RN, 1^st^ visit)]* |
|  |  | By ensuring patient understanding by providing explanation and rationale | HCP: Yeah, so that’s why it was high, it’s cause you had eaten, that uh, before the blood test. The 3 month average, the A1c is not affected, even if you eat that day because it captures 3 months of up and downs of where your blood sugar has been. *[HCP17 (RN & MD, 2^nd^ visit)]* |
|  |  | By addressing patient barriers to adhering to *MyDiabetesPlan* | Okay. If in the uh- so let’s say in 2 or 3 weeks so after the holidays if I still haven’t received the uhm uh the insulin supply, let me know. We’ll, we’ll get you some from somewhere […] ‘cause I don’t want you finding you can’t afford it and then either not buying it or, or sacrificing something else that you need because you can’t afford your insulin. *[HCP35 (MD, 3^rd^ visit)]* |
|  |  | By individualizing *MyDiabetesPlan* based on patient circumstances | HCP: So, this is talking about your physical activity, your exercise. So you said you don’t really do any at the moment.  Pt: Because due to the surgery.  HCP: ‘Cause of the surgery.  HCP: Not right now. Okay. So then we’ll just skip those for now. *[HCP248 (RPh, 1^st^ visit)]* |
|  |  | By providing encouragement and positive feedback | So your last A1c [patient’s name] was done in August of this year and you were totally at target. You had gone from 7.7 to 6.4. *[HCP39 (MD, 2^nd^ visit)]* |
|  | b) Providing information | By providing an overview of diabetes management | Um, so if we look, so looking at the plan here, this was the diabetes plan, and I, to be honest sort of blood pressure and diabetes management, they kind of both, a core component of that is the lifestyle. Right, so exercise, and the diet. *[HCP150 (MD, 3^rd^ visit)]* |
|  |  | By providing summary of health status and laboratory results | So interestingly here, you’ve actually, the past couple of years your control hasn’t been quite as good as uhm as it had been in the past. […] So I think it’s probably very timely that we look at what you can do uh to try and improve that. *[HCP133 (MD, 2^nd^ visit)]* |
|  |  | By providing hard copies of information | So this is “Cholesterol and Diabetes”, it's from the Canadian Diabetes Association, talks a little bit about what cholesterol is uhm you’ve had it tested (printer noise). It’s slightly high, here are some healthy eating tips you already agreed to. So we’ll give you this handout. *[HCP10 (RN & RD, 1^st^ visit)]* |
|  | c) Dependent on degree of clinician engagement | Engaged clinicians:  Introduce *MyDiabetesPlan* in a positive light | So this is what this is gonna tell us here, [Right] how we can make life a little bit better for you. *[HCP54 (RN, 1^st^ visit)]* |
|  |  | Summarize *MyDiabetesPlan* for the patient at end of visit | Okay. So. This is what we’re doing. So we’re gonna avoid strokes, & we’re gonna do that by exercise, okay? So, limit no more than 2 hours a day of watching TV or computer, okay? And then exercises, right? Five repetitions per set, three sets per session, three times a week. Okay? And continue to do your tai chi. Every day. Okay? So that’s the plan. And, you’ve got your husband & daughter, & me & Dr. […], & it’s very important & confident. And then we’re gonna book you for an appointment in February to come back, okay? *[HCP34 (RN, 2^nd^ visit)]* |
|  |  | Ellicit patient feedback regarding *MyDiabetesPlan* | HCP: Do you- have you found that this has helped you achieve your goals?  Pt: Yeah I- it was just re-focusing, ‘cause I, when I changed my diet my blood sugar in the morning was going too low but we didn’t want to reduce the medications [HCP: Mmm] because then we’d end up with spikes during the day [HCP: Right] so I realized I needed to eat a snack uh like uh a carbohydrate before I go to bed [HCP: Mmm] that way when I, uh you know, when I’m getting up in the morning my blood sugar is not too low. So that we changed and that’s working. *[HCP17 (MD, 2^nd^ visit)]* |
|  |  | Unengaged clinicians:  Start with his or her own agenda | Okay so, I’ll start with just reviewing how your blood, how your lab tests are…and, so your A1C is 6.9%. *[HCP17(RN & MD, 2^nd^ visit)]* |
|  |  | Return to patient's concerns only after own agenda complete | HCP: So, hold onto that.  Pt: Okay.  HCP: Okay, so sorry to interrupt you here, so nooow…  Pt: So I was—  HCP: Oh this is—  Pt: …wondering on what I can do now, is that okay? *[HCP54 (RN & RD, 2^nd^ visit)]* |
|  |  | Healthcare provider faced with time constraint | [sighs] I just said I have to get to a meeting at 2:30 so I’m gunna try and get through this. *[HCP32 (RN, 1^st^ visit)]* |
| 2) Patient’s response to *MyDiabetesPlan* | Promote open discussion | Opportunity to expresses personal fear and rationale underlying health care choices | HCP: So how important would you say it is for you to achieve your goal?  Pt: I’d would like to get 100%. I really, I really want to, I, I told her, I’m afraid to go on insulin, I don’t want to go on insulin. I know many of our sisters have it and they have a bad habit that I am afraid I would probably pick up. Cause she says well I will just put it, I will just get a little more insulin, then I will have a piece of cake. And now I was, I, that’s not a good thing. And that’s why I’m afraid to get on insulin, so I’m trying. *[Pt53 (MD, 1^st^ visit)]* |
|  | Promote patient-directed care and independence | Opportunity to direct own goal setting | Pt: I’ve actually considered there’s a Y in town, and there’s a, there’s a, I used to belong to the Y [HCP: Hmm-mm] so maybe I need some weights [HCP: Hmm] do some weights that I used to do. *[Pt57 (RN 2^nd^ visit)]* |
|  |  | Opportunity to independently complete *MyDiabetesPlan* | HCP: Uhm so I see you’ve done the, the plan, the go- the goal setting plan already it looks like?  Pt: Yes, I did it online there. *[Pt57 (RN 2^nd^ visit)]* |
| 3) Challenges to integrating *MyDiabetesPlan* into clinical care | Factors related to *MyDiabetesPlan* |  |  |
|  |  |  |  |
|  | Clinician-related factors | Reluctance to change to using *MyDiabetesPlan* | One more visit like this, & then we go back to the old way. (Laughs). *[HCP34 (RN 2^nd^ visit)]* |
|  |  | Discomfort about asking certain questions in *MyDiabetesPlan* | Um do you have a friend to support you? Some friends? [Pt: hmm actually, pauses, the family will be enough, the most important. Because they live with you]. HCP: mhmm. [Pt: so..[pauses]].  HCP: Okay so we’ll leave that one. *[HCP10a (RN & RD, 2^nd^ visit)]* |
|  |  | Open criticism of *MyDiabetesPlan* | HCP: I even tell the patients, ‘Please don’t think it’s you. Somehow it is the tool.’  HCP: So, the next one would be this long phrase and question,*[HCP19 (RD, 1^st^ visit)]* |
|  |  | Perception of lack of flexibility of *MyDiabetesPlan* | HCP So… somehow its only giving you one of the goals that you checked.  Pt: mhm.  HCP: So we checked about 5 but its only giving you..  Pt: okay.  HCP: So I guess we’ll just choose this one *[HCP17 (RN & MD, 2^nd^ visit)]* |
|  |  | Redundancy of questions in *MyDiabetesPlan* | HCP: High cholesterol, so it’s just repetitive a little bit, you know, we already answered [Yes.] these questions. [HCP19 *(RD, 1^st^ visit)*] |
|  | Patient-related factors | Patient complexity | HCP: Have you had a stroke?  PT: This is debatable because when I was hospitalized for two weeks, I was visiting my daughter, and I wanted to link with a hospital in [name of city], [Yes.] and they thought I did have a mild stroke, but they thought it didn’t show up in results.  HCP: Mhm.  PT: It was debatable whether I had or not. And then I saw a neurologist there, and she thought that it sounded like I, very late in life, I’ve become… developed MS because there were white cells, white stuff in my brain, and the spinal tap they did, and then she came up with that. So I saw a neurologist here [Yes.] in [name of city] because it was difficult for me to get up to [name of city] all the time [Yes, of course.], and she doesn’t think it was a stroke. She thinks that it was just, has some great big word, hypertension. Then my blood pressure, when I went to the hospital, my blood pressure was 220/115 or something, and I couldn’t walk, and I had all these kind of symptoms, you know [Yeah.], so whether I’ve had a stroke or not, I really can’t tell. It seems like some doctors think I have something I haven’t. *[Pt19 (RD, 1^st^ visit)]* |
|  |  | Language and/or cultural barrier | HCP: So, can you ask her to tell me what she eats?Obs: Uh, in terms of fruits or vegetables?  HCP: All of them, like what does she have for breakfast?  Obs: (Speaks to PT-34 in native language for translation.)  PT: Sometimes, uh, today is congee.  HCP: Okay.  PT: And sometimes is uh noodle. *[Pt34 (RN, 2^nd^ visit)]* |
|  |  | Inaccurate responses | Pt: Yeah The rubber band that goes like this, but not weight lifting like that.  HCP: Yeah but that’s still weight lifting! Pt-15: Yeah I do that! HCP: You didn’t tell me! [laughter]  Pt-15: Oh I thought...  HCP: You’re already doing it. Okay, so okay I check it! |
|  |  | Varied patient interpretation of questions in *MyDiabetesPlan* | When I’m able to walk, I walk everyday and I do other kinds of things but [okay] I’m not involved in any sports. I would say moderate easily. *[HCP15/16 (RD, 2^nd^ visit)]* |
|  |  | Difficulty synthesizing patient responses | How many days per week do you eat five or more servings of fruits, so again, that will be the apple and orange [Yes.] and cherries [Yes.] and berries [Yes.], and vegetables, and serving size is about 1 piece of fruit.  PT: Yes, fruit very often except I like things like cherries, so I’ll just take a little handful of cherries [Okay.] or strawberries or blueberries [Yeah.], or anything. Just a little, a handful of that, but I eat fruit every day. Vegetables [Not so much. *Laughs*], eh not so much. I mean, okay recently, because I like tomatoes, I like mushrooms, I like peppers, I like asparagus.  HCP: So if you think of a day, altogether, your vegetables, would it be adding up to a cup, like an 8-ounce measuring cup, you think?  PT: Not necessarily. *[Pt19, HCP19 (RD, 1^st^ visit)]* |
|  |  | Patient difficulty in navigating *MyDiabetesPlan* independently | Pt: So how do I go, like I tried this morning to go into it, like if I want to change…] Yeah! [P: …a goal or whatever, how do I do that?] So, were you able to log in? [Pt: I could log in, no problem.] So let me, [Pt: And my “pin/plan” (???) works and all that] good. So let me log in and “…???” [Pt: I just wasn’t sure where to click.] *[Pt262 (RPh, 2^nd^ visit)]* |
|  |  | Perception of lack of flexibility of *MyDiabetesPlan* | Pt: I just want to learn how to eat less.  HCP: Okay that’s not one of the goals on here.  Pt: Yeah I know.  HCP: But is that something that you wanna look at, it says you can select your meal… or all meals.  Pt: Uh how about reduce..  HCP: You don’t have to choose any of these, you know, you can, you don’t have to choose all of these goals. These are just the options, reviewing with you. *[Pt16, HCP17, (RN & MD, 2^nd^ visit)]* |
|  | Other factors | Technical factors external to *MyDiabetesPlan* | HCP: [clicking mouse]. I don’t know if there is a - , it won’t let me click any further. Computer error  Obs: Okay. I wonder if it is because it is on internet explorer  HCP: Ohhh  Obs:Umm, does this computer have chrome?  HCP: No. [clicking] *[HCP53 (MD, 1^st^ visit)]* |
|  |  | Challenges with logging into *MyDiabetesPlan* | HCP: -I hope I have the right person. That seems very strange.  Pt: I don’t remember saying that. I don’t know who the partner would be.  HCP: Okay, let me just take a peak here. [pause] Yeah this is you. Okay. [click] [long pause]  HCP: It’s got somebody else coming through on this. Husband and 2 adult children. *[HCP2 (RN, 2^nd^ visit)]* |
| 4) Facilitators to integrating *MyDiabetesPlan* into clinical care | Positive healthcare provider- patient relationship |  | Pt: Yeah, yeah no I have to do that and make sure I get outside at lunch time  HCP: Even if it’s a cold day, [Pt: Yeah, yeah] just go on out, take a breath, and I’m gunna  be honest with you [Name of Pt] I can sit in this chair and counsel with you, I just had lunch  in my office, [Pt: chuckles] like so I have to be honest. [Pt: Yeah, yeah] I know exactly  where you’re coming from, it’s a tough thing to do but I think there comes a point that we have  [emphasis] to [sigh] support each other and, you now I can, I can recommend that to you but I  have to also follow by example. [Pt: Yeah, yeah] So I’m gunna try and do that too. Okay? I  really need to do that myself, [Pt: Mm-hmm] so if you can do it, I should be able to do it too.  [Pt: Mm-hmm] So I can relate to that. *[Pt32 (RN, 1^st^ visit)]* |
|  | Clinician communication and synthesizing strategies | Use of open-ended questions to obtain patient responses | Okay. Alright, let’s talk about whatcha eat. So, in order to do this part I need to sorta get a sense of what you’re eating [okay] okay. [mm hmm] So tell me about your typical breakfast. *[HCP54 (RN, 1^st^ visit)]* |
|  |  | Extrapolation of answers from patient responses | And it looks like you’re spacing your carbohydrates [brief pause] throughout the day. Um, you’re, you’re not overeating and you’re, you’re sort of having carbs every time you sort of eat. [right] and even though you don’t probably have a formal lunch you do have something. [Yeah I do] So, I think you’re spacing it out. *[HCP44 (RN, 1^st^ visit)]* |
|  |  | Use of earlier questions in *MyDiabetesPlan* to facilitate later discussion | HCP: Let’s talk about your mild, moderate or vigorous activity. So again, it’s just, you know, some of it you already answered [Yes.], so mild would be the walking that you said, yoga, you know, like bowling, golfing, you know [Yes.], like leisure, swimming, or aqua fitness, those kinds of things. Do you do any of those things?  Pt: No.  HCP: But do you some walking, you said!  Pt: I walk, walk, yeah, oh yeah.  HCP: Okay. So how many days do you do?  Pt: Just about everyday. Oh well, I’d say five days a week.  HCP: Five days a week. *[HCP19 (RD, 1^st^ visit)]* |
|  |  | Helping patient interpret questions in *MyDiabetesPlan* | So are you under good control? So when I say that, most people say yes if you’re, if you’re testing and you’re getting somewhere between uh 5 and 10. *[HCP32 (RN, 1^st^ visit)]* |
|  |  | Helping patient interpret *MyDiabetesPlan* output based on previous input | HCP: So, diabetes can cause medical problems, which are preventable, and based on the information we’ve shown you 3 possible goals, okay. Losing my eyesight, avoiding a heart attack, or avoiding being on kidney dialysis. Now you need to pick one of those and we’ll then we’ll talk about some strategies to get to those.  Pt: I need to pick one?  HCP: One of these goals. Right cause you had said you wanted to avoid kidney dialysis, but prior to that you had mentioned you don’t want to lose your eyesight either. So this is what the machine has picked up and I guess it’s added avoiding having a heart attack. So one of those 3 we’d want you to pick. *[HCP44 (RN, 1^st^ visit)]* |
|  |  | Teaching patient how to use *MyDiabetesPlan* independently | HCP: If you have any, like any time you see the question mark, you can just hover your thing over it. And it’ll give you examples. *[HCP54 (RN & RD, 2^nd^ visit)]* |
|  |  | Combining *MyDiabetesPlan* use with standard of care to address patient concerns | Let’s talk about that briefly then. Are you having much to eat, snacking at night, is your dinner late?  Pt: I snacked last night but it was a piece of cheese rolled up and a piece of chicken. HCP: okay so no (emphasis) carbohydrates and do you have other snacks at night, other days?  Pt: umm, yeah I can. Not always. I’m tryna get away from it. HCP: so not consistently, but even without a snack at night you’re seeing those 9s and 10s in the morning. Pt: Yeah and I I was sinking in with stress..  HCP: And how ofte-.. how um long have you been seeing that number in the – for the fasting blood sugar? Like months, 2 months? Pt: Yeah, maybe the whole time since I’ve been away. *[HCP17,Pt16, (MD & RN, 2^nd^ visit)]* |
|  | Prior use of *MyDiabetesPlan* | Completion of *MyDiabetesPlan* by the patient prior to the appointment | HCP: Uhm so I see you’ve done the, the plan, the go- the goal setting plan already it looks like?  Pt: Yes, I did it online there. *[Pt-57 (RN, 2^nd^ visit)]* |
|  |  | Faster completion of *MyDiabetesPlan* in subsequent appointments | Good, good. Okay. So that’s all we have to do today. Okay? ‘Cause it’s much shorter. *[HCP34 (RN, 2^nd^ visit)]* |
